# Highly efficient generation of bacterial leaf blight-resistant and transgene-free rice using a genome editing and multiplexed selection system

**DOI:** 10.1186/s12870-021-02979-7

**Published:** 2021-04-24

**Authors:** Kun Yu, Zhiqiang Liu, Huaping Gui, Lizhao Geng, Juan Wei, Dawei Liang, Jian Lv, Jianping Xu, Xi Chen

**Affiliations:** Syngenta Biotechnology (China) Co., Ltd, No.25, Life Science Park Road, Beijing, 102206 China

**Keywords:** Bacterial blight, Disease, Genome editing, Transgene-free

## Abstract

**Background:**

Rice leaf blight, which is a devastating disease worldwide, is caused by the bacterium *Xanthomonas oryzae* pv*. oryzae* (*Xoo*). The upregulated by transcription activator-like 1 (UPT) effector box in the promoter region of the rice *Xa13* gene plays a key role in *Xoo* pathogenicity. Mutation of a key bacterial protein-binding site in the UPT box of *Xa13* to abolish PXO99-induced *Xa13* expression is a way to improve rice resistance to bacteria.

Highly efficient generation and selection of transgene-free edited plants are helpful to shorten and simplify the gene editing-based breeding process. Selective elimination of transgenic pollen of T0 plants can enrich the proportion of T1 transgene-free offspring, and expression of a color marker gene in seeds makes the selection of T2 plants very convenient and efficient. In this study, a genome editing and multiplexed selection system was used to generate bacterial leaf blight-resistant and transgene-free rice plants.

**Results:**

We introduced site-specific mutations into the UPT box using CRISPR/Cas12a technology to hamper with transcription-activator-like effector (TAL) protein binding and gene activation and generated genome-edited rice with improved bacterial blight resistance. Transgenic pollen of T0 plants was eliminated by pollen-specific expression of the α-amylase gene *Zmaa1*, and the proportion of transgene-free plants increased from 25 to 50% among single T-DNA insertion events in the T1 generation. Transgenic seeds were visually identified and discarded by specific aleuronic expression of DsRed, which reduced the cost by 50% and led to up to 98.64% accuracy for the selection of transgene-free edited plants.

**Conclusion:**

We demonstrated that core nucleotide deletion in the UPT box of the *Xa13* promoter conferred resistance to rice blight, and selection of transgene-free plants was boosted by introducing multiplexed selection. The combination of genome editing and transgene-free selection is an efficient strategy to accelerate functional genomic research and plant breeding.

**Supplementary Information:**

The online version contains supplementary material available at 10.1186/s12870-021-02979-7.

## Background

The use of new breeding techniques, such as genome editing, has been extended beyond genetically modified (GM) input-trait products and expanded for the commercialization of genome-edited output-trait products [1]. Genome editing has numerous advantages over previous technologies, most significantly in that it allows targeted, single-gene mutations across the entire plant genome [1]. The alteration of a specific DNA locus without leaving behind heterologous genetic elements offers a significant advantage of this system over traditional genetic modification approaches.

Rapidly and efficiently generating transgene-free, edited plants is critical for breeders to shorten the gene editing-based breeding process. The presence of editing machinery components increases the difficulty of assessing the heritability and phenotypic stability of target-edited plants. The generation of new mutations by residual editing machinery makes it difficult to interpret the inheritance of mutant genotypes. Moreover, the chance of off-target mutations is also increased [2] and may lead to regulatory concerns related to genetically modified organisms [3]. Transgene-free plants can be obtained by segregation of progeny in subsequent generations and identified based on molecular detection. However, these methods are expensive, laborious and time consuming. Several strategies have been reported for screening or enriching transgene-free progeny. Specific expression of the fluorescent protein mCherry in *Arabidopsis* seeds was used for visual selection of edited progeny [4]. Another strategy for selection of transgene-free plants was to incorporate an editing vector with an RNAi expression cassette that silences the herbicide resistance gene CYP81A6 encoding a P450 cytochrome protein. This strategy enables the isolation of bentazon-resistant transgene-free plants from susceptible transgene plants by using herbicides [5]. Although these strategies greatly accelerated the selection of transgene-free plants, they did not increase the proportion of desired transgene-free plants in the population. Recently, He et al. reported a new strategy for transgene-free isolation via a programmed self-elimination system (transgene killer CRISPR, TKC) that actively and automatically eliminated any plants that contained the T-DNA insertion [2]. The bacterial *barnase* gene driven by the embryo-preferred promoter REG2 and the rice ORFH79 gene driven by the CaMV 35S promoter were used to kill any embryos and male gametophytes, respectively, that contained the transgenes. This method is effective and thorough; on the other hand, it also leads to a high risk of recovering a low number of edited events. Desirable edited transgenic plants may also lose the chance of being selected when transgene components can be removed by segregation of progeny. In addition, using rice ORFH79 to kill male gametes limits the application of TKC among different crops. Compared with killing embryos and male gametophytes, it is preferable to kill only transgenic male gametophytes, which not only reduces the risk mentioned above and increases the proportion of transgene-free offspring, but also can prevent the spread of transgenic pollen. Choosing components that are common to monocotyledons or dicotyledons can improve the efficiency of the system. The α-amylase gene *Zmaa1* is a potential candidate that has been applied successfully in seed production technology (SPT) to kill transgenic pollen by disrupting starch accumulation during transgenic pollen maturation, which deprives the necessary energy source for fertilization [6]. Other pollen inactivation genes, such as *barnase* and *E. coli* DNA (Adenosine-N6-)-Methyltransferase (DAM)*,* have been used to deliver male sterility in diverse plants [7–11]. Therefore, these genes can be used as potential components for the construction of gene editing vectors to kill transgenic pollen in diverse plant species and increase the proportion of nontransgenic offspring. Furthermore, a visual marker, such as *DsRed* or *mCherry,* can also be used together with the pollen-killer cassette so that the nontransgenic progeny can be distinguished from the transgenic progeny by simple visual inspection.

Rice bacterial leaf blight disease caused by *Xanthomonas oryzae* pv. *oryzae* (*Xoo*) resulted in severe yield losses, especially in Asia and Africa [12, 13]. Approximately 42 genes conferring resistance against various races of *Xoo* have been identified in both cultivated and wild relatives of rice [14]. The *xa13* gene encoding a plasma membrane protein is fully recessive and confers resistance to *Philippine Xoo* race 6 (strain PXO99) [15]. The resistant allele of the *xa13* gene differs from its dominant (susceptible) allele in the coding region by only one amino acid residue; the 238th residue (alanine) of the dominant XA13 protein is replaced by threonine in the recessive xa13 mutant protein [16]. However, disease resistance was not conferred by amino acid residue variation but by alterations in the promoter regions, which caused differences in expression between the resistant and susceptible alleles [15, 16]. Compared with the dominant alleles, all recessive alleles had deletions, mutations and substitutions in the corresponding regions from promoter sequence positions − 86 to − 69, suggesting that promoter mutations may result in xa13-mediated disease resistance [15]. By analyzing the gene expression level driven by truncated and mutated *xa13* promoters in rice, it has been indicated that the promoter of *Xa13* harbors an upregulated by transcription activator-like 1 (UPT) effector box, which is the only PXO99-responsive *cis*-regulating element in the activation of *Xa13* expression, and the 5′-terminal second, third and fourth nucleotides of the box are the binding sites for the bacterial protein [17, 18]. Li et al. reported that a 149-bp deletion in the UPT of the *Xa13* promoter deprived bacterial blight of its ability to induce *Xa13* gene expression and conferred race-specific resistance [19]. The identification of UPT boxes promoted the application of genome editing technology to knock out key regions and confer resistance.

**Fig. 1:**
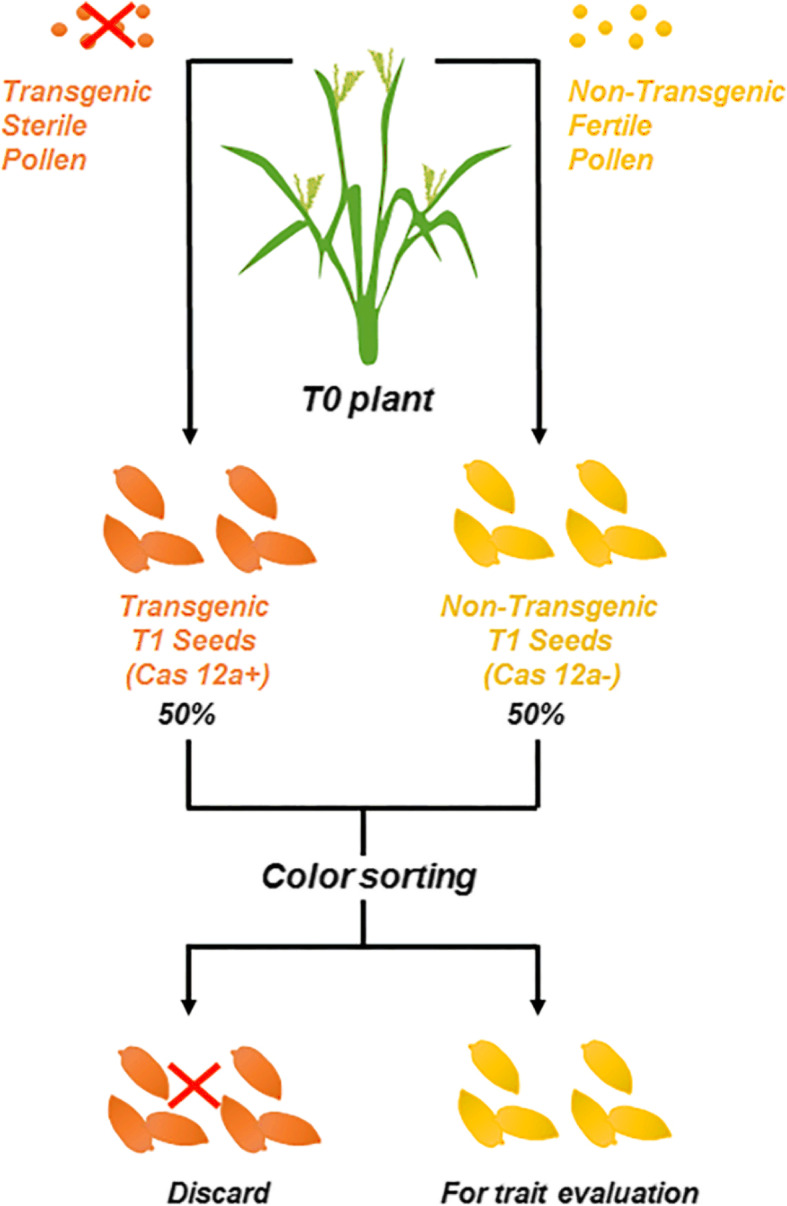
Schematic diagram of the strategy for transgene-free plant enrichment and selection. Increasing the proportion of nontransgenic offspring by hampering T-DNA transmission in male gametophytes and sorting nontransgenic seeds by using a visual selection marker. For single T-DNA insertion lines, self-pollination of the resulting T1 propagates 50% of the transgenic seeds and 50% of the nontransgenic seeds, indicating that the proportion of desired T1 seeds will be increased from 25 to 50%. For multicopy T-DNA insertion lines, the T-DNA-free progeny segregation rate will increase 2n (2 to the power n) times. N is equal to the copy number

Here, we report a method to generate *Xoo*-resistant rice by CRISPR/Cas12a-mediated targeting of the binding site of the UPT box in the *Xa13* promoter region and further demonstrate critical nucleotides (5′-terminal second, third and fourth nucleotides) for transcription-activator-like (TAL) effector protein binding and *Xa13* activation. We improved transgene-free plant selection by applying a GM pollen elimination and dsRed marker selection system, which greatly enriched the proportion and selection efficiency for transgene-free edited progeny (Fig. 1). The streamlined procedure demonstrated its utility in genome editing for plant breeding and basic research with improved process efficiency.

## Results

### Generation of LbCas12a transgenic rice plants and selection of events with edits in the UPT of the Xa13 promoter

**Fig. 2:**
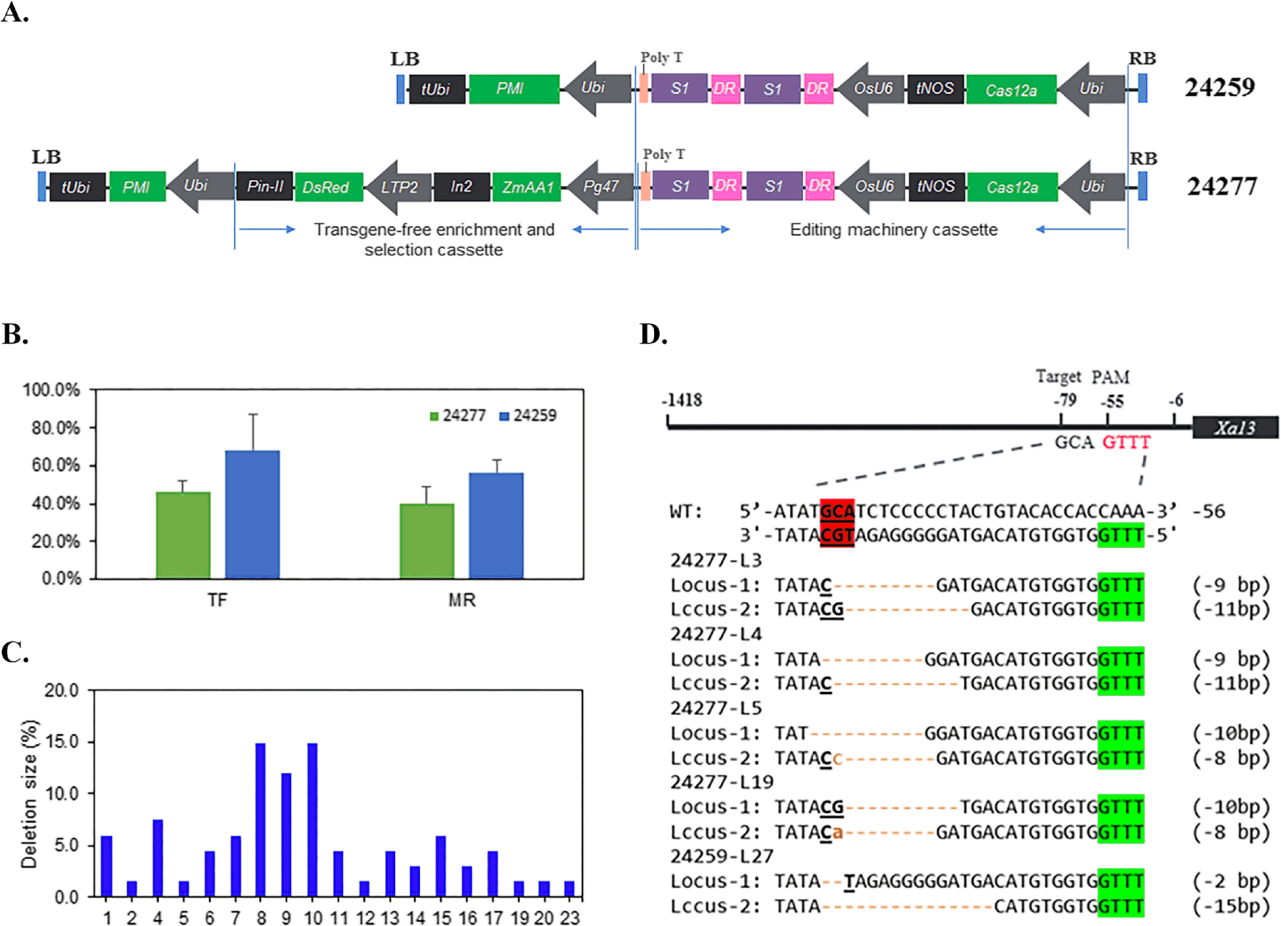
Generation of Xa13 promoter mutants using CRISPR/Cas12a editing vectors. **a** Schematic of the T-DNA region of the editing vectors 24259 and 24277. **b** Comparison of transformation frequency between two vectors. **c** Deletion sizes derived by LbCas12a in 67 T0 lines from two constructs; bp, base pairs. **d** Different deletions occurred at the expected target sites, that is, the 5′-terminal second, third, and fourth nucleotides of the UPTPthXo1 box in the Xa13 promoter in the 5 T0 events that were selected as candidates for further resistance evaluation

To confer *Xoo* resistance to rice, we constructed two LbCas12a plasmid vectors harboring a 24-nt crRNA to target the UPT box of the rice *Xa13* (DQ421395.1) promoter, which is the binding site for the bacterial TAL effector protein. The core target site ‘GCA’ located at the − 79 position of the *Xa13* promoter is 24 bp away from the TTTV protospacer-adjacent motif (PAM) motif downstream on the opposite strand (Fig. 2d). A multiplexed selection system was used to increase the proportion of and to select T-DNA-free plants in the T1 generation. The multiplexed system consists of pPG47::Bt1:Zm-aa1 and pLTP2::DsRed expression cassettes. pPG47 is the promoter of maize polygalacturonase gene, which specifically expressed in late-developed pollen and pLTP2 is cloned from the endosperm-preferred lipid transfer protein (LTP2) gene in barley [6]. To measure the effectiveness of the system, a construct, 24277, was created that contained both *Zmaa1* and *DsRed* expression cassettes; another construct, 24259, was used as a control (Fig. 2a). Constructs were introduced into the indica rice variety IR58025B via *Agrobacterium*-mediated transformation. The T-DNA copy number was measured, and mutations were identified by the TaqMan assay and then confirmed by colony sequencing. The transformation frequency (TF, number of positive events/number of infected explants) and mutation rate (MR, number of mutant events/number of positive events) of constructs 24277 and 24259 based on the results of three experiments were investigated (Table 1 and Fig. 2b). The TF and MF of the *Xa13* promoter targeted by LbCas12a were 46.1% (227/492) and 39.6% (90/227), respectively, for 24277 and 68.3% (314/460) and 56.4% (177/314), respectively, for 24259. Both the TF and MR of 24277 were lower than those of 24259. The main possible reason of lower TF may cause by bigger construct size, 24277 is 24 kb, 8 kb bigger than 24259. Multi-copy event ratio (83.09%) of 24259 is more than that (73.26%) of 24277, which explains higher MR and mimics similar finding in other studies in Syngenta. The range of deletion sizes derived by LbCas12a in 42 mutants ranged from 1 to 23 bp, and the majority of the deletions ranged from 8 to 10 bp in size (Fig. 2c and Supplementary Table 1). Forty-four single T-DNA insertions and backbone-free T0 events with three core nucleotide deletions in the *Xa13* promoter from the two constructs were chosen to produce T1 seeds for further evaluation (Fig. 2d).

**Table 1:** Transformation frequency and mutation rate of two constructs

		Explants #	Positive #	TF	Mutants	Mutant rate
**Independent transformation event**	24277–1	168	59	35.1%	16	27.1%
	24277–2	100	39	39.0%	16	41.0%
	24277–3	224	129	46.1%	58	45.0%
	24259–1	168	68	40.5%	32	47.1%
	24259–2	100	76	76.0%	42	55.3%
	24259–3	192	170	68.5%	103	60.6%
**Summary**	24277	492	227	46.10%	90	39.6%
	24259	460	314	68.30%	177	56.4%

### The nontransgenic rate in progeny was increased by GM pollen elimination, and nontransgenic seeds were identified visually through the expression of color marker genes

T-DNA segregation in T1 offspring of 10 single-copy T-DNA insertion T0 events from the two vectors was analyzed by the TaqMan qPCR assay and RFP expression. As shown in Table 2, most T1 plants from 24259 events harbored the T-DNA transgene, which was no longer needed, and the proportion of transgene-free progeny plants ranged from 19.68 to 29.65%. In contrast, the proportion of transgene-free plants from 24277 ranged from 46.92 to 50%, and the ratio between transgene and transgene-free plants was close to 1:1. The results indicated that the expression of *Zmaa1* resulted in sterility of transgenic pollen, which led to an increase in the proportion of transgene-free offspring.

**Table 2:** Genetic analyses of T-DNA segregation in the T1 population of two constructs

Event ID	Construct	Detection of RFP expression						Taqman assay						
		No. seeds RFP+	No. seeds RFP-	Transgene-free %	Observed ratio	Expected ratio	χ2	T-DNA +	T-DNA -	Transgene-free %	Observed ratio	Expected ratio	χ2	Ave. accuracy rate of visual selection
**L18**	24277	103	97	48.50%	1: 0.94	1: 1	0.18	69	61	46.92%	1: 0.88	1: 1	0.49	98.62%
**L31**		98	103	51.24%	1: 1.05	1: 1	0.12	96	101	51.27%	1: 1.05	1: 1	0.13	
**L33**		98	102	51.00%	1: 1.04	1: 1	0.08	96	101	51.27%	1: 1.05	1: 1	0.13	
**L5**		98	103	51.24%	1: 1.05	1: 1	0.12	93	99	51.56%	1: 1.06	1: 1	0.19	
**L4**		93	107	53.50%	1: 1.15	1: 1	0.98	92	106	53.54%	1: 1.15	1: 1	0.99	
**L8**	24259							146	44	23.16%	3.30: 1	3: 1	0.34	N/A
**L21**								148	48	24.49%	3.08: 1	3: 1	0.03	
**L22**								151	37	19.68%	4.08: 1	3: 1	2.84	
**L26**								143	51	26.29%	2.80: 1	3: 1	0.17	
**L34**								140	59	29.65%	2.37: 1	3: 1	2.29	

**Fig. 3:**
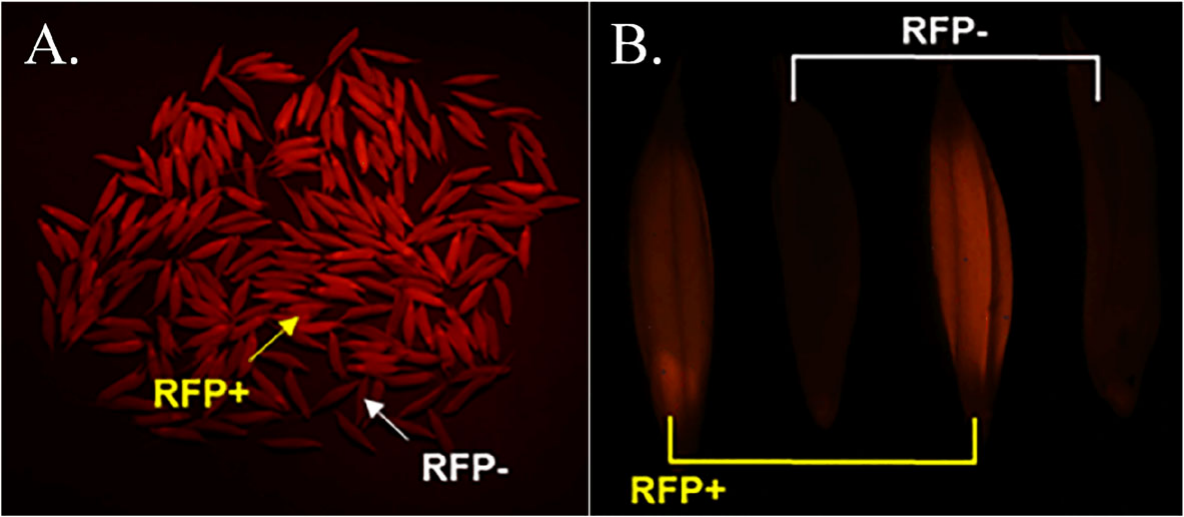
Visual sorting of T1 seeds by fluorescence detection. The T-DNA-free seeds did not produce red fluorescence. **a** Visual difference of the T-DNA-free population and T-DNA insertion population by fluorescence detection. **b** No red fluorescence was detected in T-DNA-free seeds

Normally, transgene-free plants are identified by PCR or other molecular tests, such as the TaqMan assay. However, the traditional methods are laborious and inefficient, and transgene-free plants are only identified after seed germination. For fast and efficient detection of transgene-free seeds, RFP was expressed under the control of an endosperm-preferred promoter, and transgene-free plants could be visually identified using a fluorescence detector (Fig. 3). The *Zmaa1* and *DsRed* expression cassettes and other transgene components were located within the same T-DNA and thus were tightly linked and cosegregated. Therefore, the T1 seeds of 24277 displaying strong red fluorescence were transgenic seeds and easy to distinguish from transgene-free seeds that did not show any fluorescence. To verify the accuracy of this visual detection method, these T1 seeds were germinated and tested by the TaqMan assay. As shown in Table 2, the average accuracy rate of visual detection reached 98.62%, confirming that the approach is highly effective for the identification of transgene-free progeny seeds.

### Core nucleotide deletion of UPTPthXo1 in the Xa13 promoter results in resistance to PXO99

**Fig. 4:**
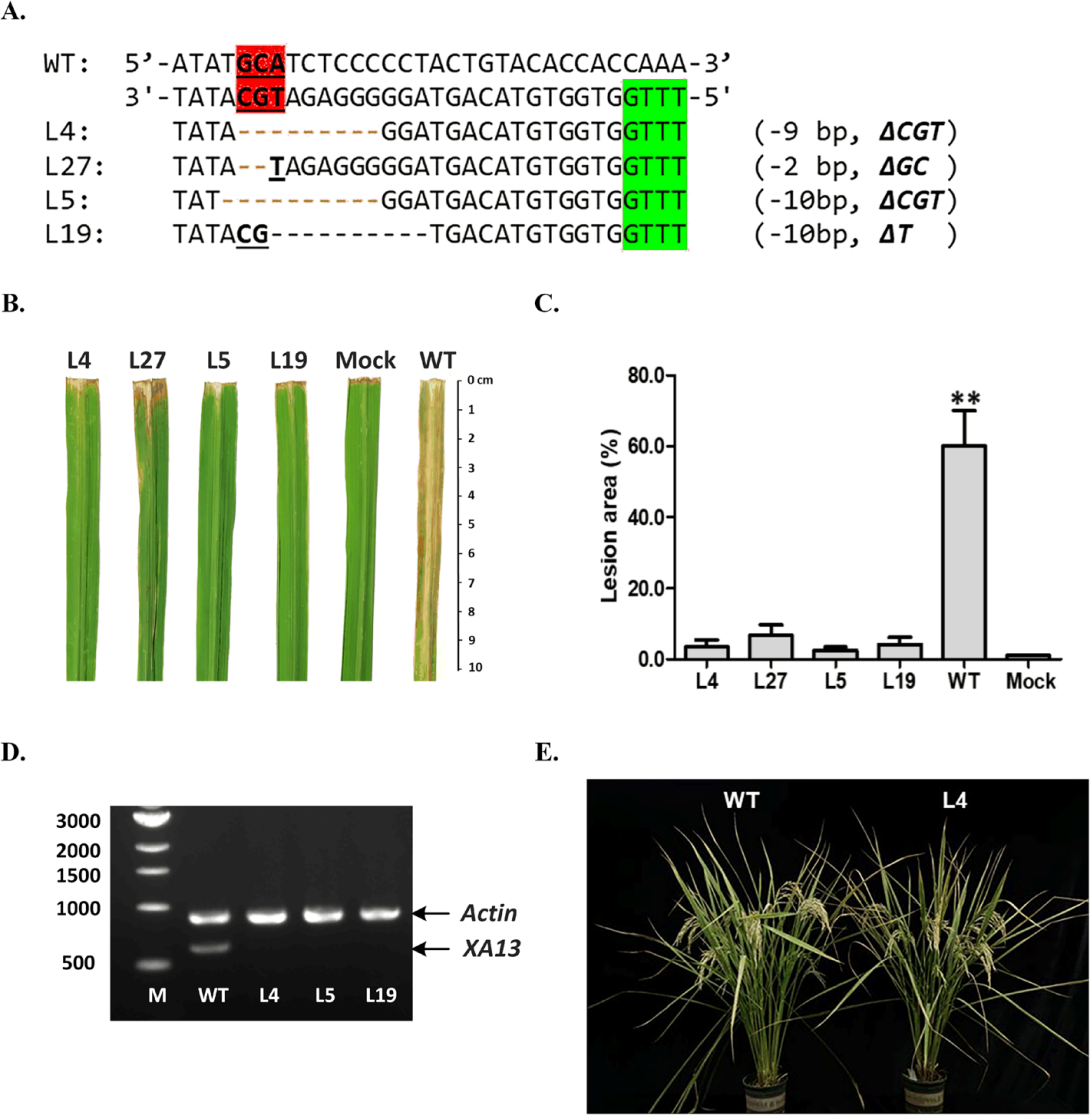
Targeted mutation of the Xa13 promoter to enhance resistance to bacterial blight. **a** Homozygous mutagenesis in the *Xa13* promoter of four T1 lines selected for resistance evaluation. **b** Leaves of WT and mutant lines at heading stage showing resistance or susceptibility to bacterial blight at the heading stage. Leaves were detached from the inoculated plants at 14 dpi for photography. **c** Lesion area of wild type and mutant lines 14 days after inoculation. WT, wild type; Mock, without pathogen. The asterisk (**) indicates that a significant difference (*P* < 0.01, Student’s t test) was detected between mutant lines and wild-type plants. **d** Expression of the *Xa13* (DQ421395.1) gene determined by RT-PCR in the leaves of wild-type and mutant lines 72 h after PXO99 infection. The *OsActin* gene (AY212324) was used as an internal control. **e** Morphology of wild-type and mutant lines. The gel images in (**d**) were cropped; the original gel images are shown in Supplementary Fig. 1

To characterize the resistance to bacterial leaf blight, four homozygous mutant T1 lines, namely, L4, L5, L19 and L27, with deletions in one, two or all three core nucleotides of the UPT box (Fig. 4a), were chosen for infection with the Philippine *Xoo* race 6 (strain PXO99) at the heading stages, and the relative expression level of the *Xa13* gene was determined by RT-PCR in the leaves of wild-type and mutant lines 72 h after PXO99 infection. The mutant lines showed significant resistance to the PXO99 strain (Fig. 4b), with an average lesion area of 2.4 ± 0.8% to 6.7 ± 3.1% compared with 60.0 ± 9.9% for the wild control IR58025B (Fig. 4c and Supplementary Table 2). Expression of the *Xa13* gene in mutant lines was not detected, which showed that deletion of core nucleotides in the UPT box resulted in the *Xa13* gene losing its ability to be induced by PXO99 (Fig. 4d). Among mutant lines with various deletions of three core nucleotides, there was no obvious difference in lesion area (*P* < 0.001, data not shown). The results show that these three nucleotides of the UPT box are important for bacterial TAL effector protein binding and gene activation. The result showing that mutations in any one of these sites abolished PXO99-induced gene expression and conferred race-specific resistance is consistent with previous reports [17, 19]. No negative agronomic phenotype, such as a decrease in fertility or seed setting rate, was observed in the mutant lines with targeted edits in the *xa13* promoter (Fig. 4e).

## Discussion

Genome editing has become a powerful tool that allows breeders to improve the agronomic traits of crops in plant breeding [20]. The primary objective of genome editing is to obtain transgene-free edited plants or seeds with desired trait performance. In selfed progeny, only approximately 25% of the T1 plants from a single copy T-DNA insertion T0 line are transgene-free, and approximately 75% of the workload is wasted on analyzing the undesirable transgenic plants. Increasing the seed number of edited, transgene-free progeny in the T1 generation can significantly increase the chance of selecting desirable edited lines and therefore shorten the breeding cycle. He et al. reported a highly efficient way to eliminate any T1 plants that contained the CRISPR/Cas9 system, and the TKC system killed any embryos and male gametophytes that contained the transgenes; however, this system is similar to a double-edged sword since it introduces risks while being efficient. CRISPR-mediated genome editing efficiency varies significantly among different species and even among different tissues of the same plant species [21]. In particular, the low efficiency of precise gene editing in plants, including DNA fragment knock-in and gene replacement, remains a major challenge [22]. Precious plants that contain desired edits but still contain the T-DNA component will be killed by TKC, instead of the component being retained to be removed by segregation. Therefore, in our research, we increased the proportion of transgene-free offspring by only hampering with the transmission of T-DNA in male gametophytes without interference in female gametes, and an additional benefit was prevention of the spread of transgenes through pollen.

A transgene-free selection system can be used as the common component of the editing base vector to increase the efficiency of the editing pipeline. The rice *ORFH79* gene limits the application of the TKC system in different crops. Devitalization of transgenic pollen by *Zmaa1* under the PG47 promoter has been applied successfully in some monocot crops, such as corn, rice and wheat [6, 23, 24]. Therefore, this cassette is an ideal candidate as a base component of a monocot crop-editing vector. To extend the application of the transgene-free selection system in dicotyledonous crops, we will further test another anther tapetum-specific promoter, TA29, to drive the expression of pollen-lethal genes.

Since T-DNA transmission in female gametophytes is allowed in our system, a selection component was included to distinguish transgenic progeny from nontransgenic progeny. Expression of the *DsRed* gene under the endosperm-preferred promoter LTP2 was used to select transgene-free seeds visually, which is very convenient and efficient. This visual sorting cassette was successful in corn and rice seeds but failed in soybean seeds because of the thick seed coat.

We generated transgene-free T1 plants resistant to infection by the bacterial pathogen PXO99 by mutating the target site of the *xa13* gene promoter with CRISPR/Cas12a. In contrast to Li’s report, in which bacterial resistance in rice was conferred by a 149-bp deletion in the *Xa13* promoter generated by a double-sgRNA site-directed mutation, we achieved this effect by deleting only two core nucleotides of UPTpthxo1 in the *Xa13* promoter (L27). We also verified that absence of any of the 5′-terminal second, third, and fourth nucleotides of the UPTPthXo1 box can confer race-specific resistance, providing direct evidence to support the conclusion in Yuan’s report [17]. Furthermore, the mutant plants with disease resistance did not show sterility or decreased yield.

## Conclusions

We developed a transgene-free plant enrichment and selection method to generate bacterial pathogen-resistant transgene-free rice. This method can be applied to accelerate the breeding of genome-edited materials.

## Materials and methods

### Vector construction

The rice codon-optimized Cas12a from *Lachnospiraceae bacterium* ND2006 containing two nuclear localization signals (NLSs) at its N- and C-termini were as described in a previous report except for 3-bp changes made to remove 2 Bsp119I sites and one RsrII site [25]. This gene was driven by the sugarcane ubiquitin 4 promoter (prSoUbi4) in two binary vectors. A tandem duplicate crRNA array (DR-S1-DR-S1) driven by the OsU6 promoter was designed as described by Wang et al. [26]. A 9-bp poly (T) short sequence was used to terminate the crRNA (OsU6-DR-S1) expression cassette. Compared to the base vector 24259, two extra expression cassettes were present in the test vector 24277.

### Plant transformation and plant nursery

IR58025B rice was used for transformation. *Agrobacterium*-mediated transformation was performed according to a protocol reported previously [27]. PMI-positive plants were identified via a selection medium containing mannose [28]. Surviving plants were subjected to a TaqMan assay to check the T-DNA copy number and target sequence mutation in the *Xa13* promoter. Plants with single-copy T-DNA insertion, backbone-free and target site mutations in the *Xa13* promoter were sent to the greenhouse. Plants were grown in greenhouse in 170 × 150-mm pots, each filled with turf, peat moss and nutrient-rich soil at a ratio of 3:2:1 plus 40 g of Osmocote, a 3–4 month controlled-release fertilizer (17–7-12). Watering was managed via drip irrigation. The growth conditions were 30 ± 2 °C in the day and 25 ± 2 °C at night, and the photoperiod was set to 12 h of day and 12 h of night.

### TaqMan assay and sequence analysis of targeted mutations

TaqMan quantitative PCR (qPCR) assay was performed to determine the T-DNA copy number and target sequence mutation. Genomic DNA was extracted from leaves by following the protocol for the Promega Magbeads Plant Genome Extraction Kit. Gene-specific primers/probes were designed using PrimerExpress3.0 software and synthesized by Life Technologies. Real-time (RT) qPCR was performed in an ABI 7900HT real-time PCR system. Each 10-μl real-time PCR contained 5 μl of 2x Sigma JumpStart Master Mix (Sigma-Aldrich Corporation), 3 μl of DNA, 0.2 μl of 50x TaqMan assay stock solution (final concentration: 300 nM for primers and 100 nM for probe) and 1.6 μl of water. The real-time PCR conditions were as follows: 95 °C for 5 min; 40 cycles of 95 °C for 5 s followed by 60 °C for 30 s. The data were analyzed using the SDS 2.4 software.

The candidates screened by the TaqMan assay were further confirmed by colony sequencing. The targeted regions were amplified with KOD-PLUS-Neo (Toyobo) and cloned into the pEASY vector (pEASY-Blunt Zero Cloning Kit, Transgen). Ten independent random clones were selected for Sanger sequencing (Life Technologies). The sequences were aligned to the wild-type sequence in Vector NTI.

### Seed color sorting

A fluorescence detector was used for seed color sorting. With matching barrier filter glasses, the red fluorescent seeds could be easily sorted from the nonred fluorescent seeds. The number of red fluorescent and nonred fluorescent seeds in different T1 events was counted manually.

### Pathogen inoculation

*Xoo* strain PXO99 was cultivated on petri dishes with YDC medium. Bacterial pathogens were scraped from the petri dishes to make suspensions at 5 dpi with ddH_2_O, and the suspension concentration was adjusted to OD 1.0 for inoculation. Rice plants were inoculated by the leaf clipping method at the booting stage, and the control plants were treated with ddH_2_O [29]. Inoculated plants were incubated in a growth chamber at 25 °C in darkness for 24 h and then transferred to normal rice plant growth conditions. Lesion length and area were measured at 14 days post inoculation, and the mean and standard deviation were calculated to analyze *Xoo* resistance.

### Gene expression analyses

Total RNA from the leaves, anthers, and roots of rice was extracted with the RNAprep Pure Plant Kit (Tiangen Biotech, www.tiangen.com); 1 μg of total RNA was used for cDNA synthesis using the Superscript III First-Strand Synthesis System (Invitrogen) and the oligo-dT primer. Semiquantitative RT-PCR was conducted as described by Zhou et al. [30]. The PCR primers for *Xa13* were 5′-ATGGCAGGAGGTTTCTTGTCC-3′ and 5′-AAGAAGCCGCCCACGTTC-3′. The primer sequences for the *OsActin* (AY212324) control gene were 5′-GCAGAAGGATGCCTATGTTG-3′ and 5′-GGACCCTCCTATCCAGACAC-3′.

## Supplementary Information

**Additional file 1: Supplementary Table 1.** Sequence analysis of mutations in the Xa13 promoter of T0 lines.

**Additional file 2: Supplementary Table 2.** Raw data and ANOVA analysis of lesion length and area of mutant lines and the WT after infection.

**Additional file 3: Supplementary Fig. 1.** Original gel picture of Xa13 gene expression determined by RT-PCR

## Data Availability

All data generated or analyzed during this study are included in this published article (and its additional files). Materials are not publicly available due to patent and licensing limitations.

## Abbreviations

XOO: *Xanthomonas oryzae* pv. *oryzae*
UPT: Upregulated by TAL effector
TAL: Transcription-activator-like effector
GM: Genetic modification
TKC: Transgene killer CRISPR
SPT: Seed production technology
PAM: Protospacer-adjacent motif
TF: Transformation frequency
MR: Mutation rate
RFP: Red fluorescent protein
LTP: Lipid transfer protein
RT-PCR: Reverse transcription-polymerase chain reaction
