# Supplementary material for: Highly efficient generation of bacterial leaf blight-resistant and transgene-free rice using a genome editing and multiplexed selection system
Source: BMC Plant Biol. 2021 Apr 24;21:197. doi: 10.1186/s12870-021-02979-7 (PMC8066475; doi:10.1186/s12870-021-02979-7)
Supplement: Supplementary file 3 — Additional file 3: Supplementary Fig. 1. Original gel picture of Xa13 gene expression determined by RT-PCR [file 12870_2021_2979_MOESM3_ESM.pdf]

Highly efficient generation of bacterial leaf blight-resistant and transgene-free rice using a genome editing and multiplexed selection system

Kun Yu[1], Zhiqiang Liu[1], Huaping Gui[1], Lizhao Geng[1], Juan Wei[1], Dawei Liang[1], Jian Lv1, Jianping Xu[1] & Xi Chen*

[1]Syngenta Biotechnology China Co. Ltd, 25 Life Science Park Road, Beijing 102206, P.R. China

***Xi.Chen@syngenta.com**

Original gel picture of Xa13 gene expression determined by RT-PCR

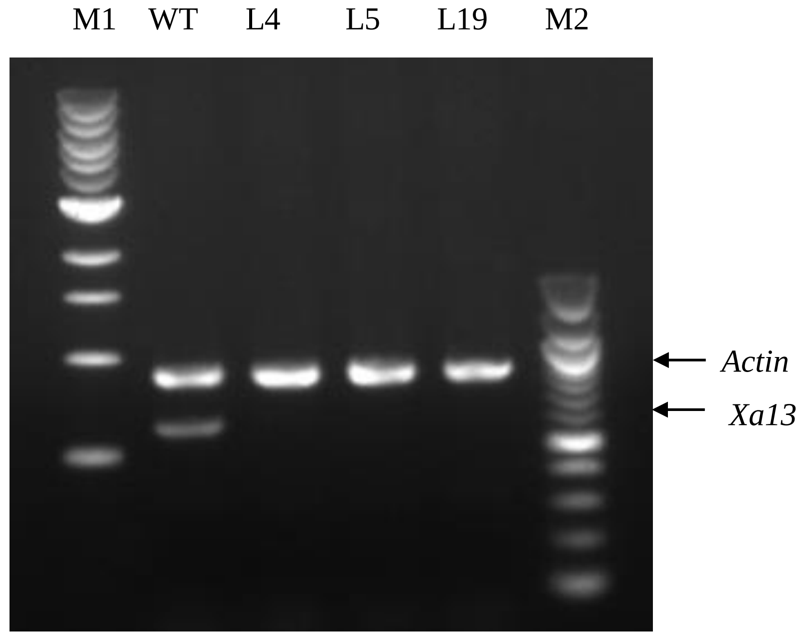

M1: 1 Kb ladder; M2: 100 bp ladder

Supplementary Figure 1
